# MDT: A simple tool to facilitate dropcasting on *in situ* TEM MEMS chips

**DOI:** 10.1016/j.ohx.2025.e00666

**Published:** 2025-06-21

**Authors:** Matthias Quintelier, Joke Hadermann

**Affiliations:** EMAT, Department of Physics, University of Antwerp 2020 Antwerp, Belgium

**Keywords:** *In situ*, TEM, Sample preparation, Dropcasting

## Abstract

*In situ* transmission electron microscopy with holders based on the use of micro-electromechanical systems provides valuable insights into material kinetics, yet sample preparation remains complex due to fragile silicon nitride (Si_3_N_4_) windows and the risk of leaks and contamination during dropcasting. This results in failed experiments and increases experimental costs. This paper introduces the MEMS Dropcasting Tool (MDT), a cost-effective, in-house 3D-printable solution that confines the droplet to a specific area, preventing particle migration under the O-ring and reducing contamination risks at the inlet and outlet ports of closed cell chip holders.

## Graphical abstract

**Figure:**
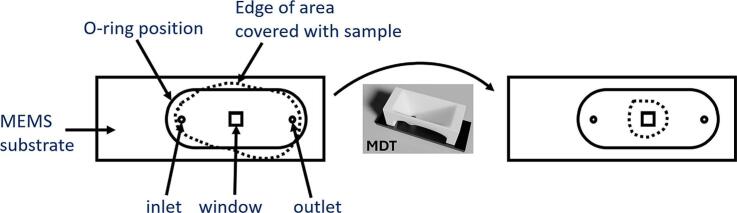

Specifications table

**Table:** 

Hardware name	MDT
Subject area	• Engineering and materials science
Hardware type	• Mechanical engineering and materials science.
Closest commercial analog	No commercial analog is available.
Open source license	CC BY-NC 4.0
Source file repository	https://doi.org/10.5281/zenodo.15667094
Cost of hardware	Cost depending on the 3D printer and chosen material, but roughly € 0.25.

## 1 Hardware in context

*In situ* transmission electron microscopy (TEM) has seen significant advancements over the past several decades and has proven essential for gaining insights into material kinetics under both elevated [[1], [2], [3], [4]] and cryogenic [[5], [6], [7], [8]] temperatures. Initially, *in situ* TEM holders used conventional TEM grids for heating and cooling, however, modern *in situ* TEM holders now incorporate micro-electromechanical systems (MEMS) chips. These chips are generally made of silicon and contain small electron-transparent silicon nitride (Si_3_N_4_) windows.

The thickness of these windows varies by manufacturer, but to achieve optimal resolution, they are often made as thin as possible, typically between 20 nm and 50 nm. In addition, specialized *in situ* holders are now available that enable the introduction of gases and liquids for detailed observations of nanoscale dynamics [[9], [10], [11], [12], [13], [14]].

**Fig. 1:**
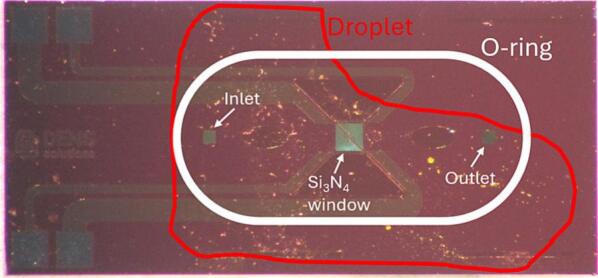
Picture of a MEMS chip with a 2 µl droplet of an ethanol/MOF-74 solution dropcasted on it. The droplet size, the inlet/outlet holes, the Si_3_N_4_ window, as well as the O-ring position are indicated on the picture.

During conventional sample preparation on such MEMS chips for gas phase and liquid phase experiments, a small volume of the sample, dissolved in a suitable solvent, is dropcasted onto the Si_3_N_4_ windows of the bottom MEMS chip. An O-ring is then placed between the top and bottom chips to create a sealed nanoreactor. However, achieving accurate dropcasting is challenging, often resulting in uneven particle distribution, with particles missing the Si_3_N_4_ windows, contaminating the inlet and outlet holes, or settling under the O-ring, which compromises the seal. (Fig. 1) These issues can lead to leaks, damage the O-ring, contaminate the inlet or outlet and cause, oppositely, also the absence of particles on the windows themselves. Each of these issues results in a failed experiment. The thinness of the Si_3_N_4_ windows, while beneficial for resolution, also makes them fragile and prone to breakage, so any correction that requires the user to re-extract the holder, repeat the dropcasting process, and reassemble the setup increases the risk of breaking the window.

To limit droplet spread, solvents with higher surface tension, such as water, can be used. However, many materials, including MOFs, halide perovskites, and alkali metal salts, are water-sensitive and cannot be dissolved in it.

Literature describes techniques to avoid drying-artifacts during dropcasting [15], as well as various alternative techniques for sample preparation on MEMS devices for *in situ* TEM studies instead of dropcasting. However, these methods often involve complex equipment such as aerosol jet printers [16], nanocapillaries [17], double shadow masks [18], pulsed lasers [19], micromanipulators [20] or FIB [21], which may not be suitable for all materials or solvents and are not always readily available in each research laboratory.

## 2 Hardware description

**Fig. 2:**
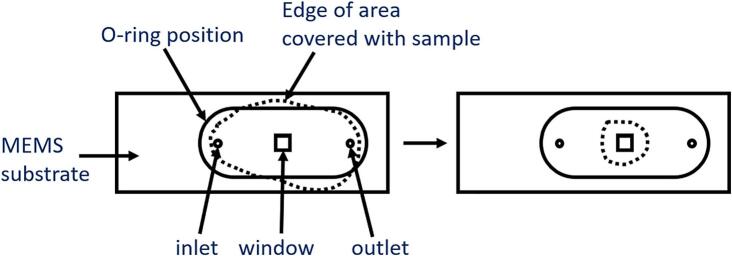
Schematic illustration of the aim of the MDT.

**Fig. 3:**
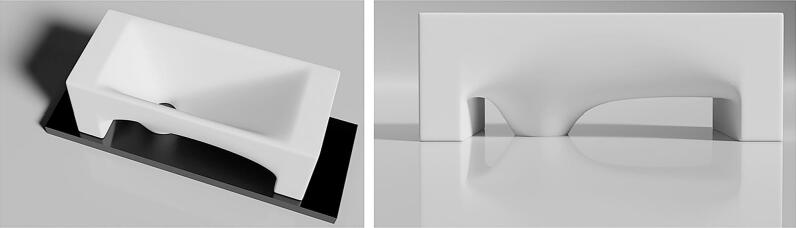
3D model for the MDT.

The aim of the MDT was to confine the dropcasted solution—regardless of the solvent—onto the MEMS chip, specifically on the electron-transparent viewing windows and within the area sealed by the O-ring (see Fig. 2). To achieve this, we designed a funnel-like structure that can be placed over the MEMS chip (Fig. 3). During dropcasting, this tool guides the droplet to the desired region and contains it while the solvent evaporates, leaving the particles precisely on the viewing window. As the tool is ideally used as a consumable to avoid cross-contamination between experiments, we tested the possibility to manufacture it at low cost using 3D printing.

The model shown in Fig. 3 was developed in Blender, the files are available in the Supplementary information and can be freely adapted further to specific needs and specific chips. The 3D printing allows to use a material of choice, however, as demonstrated below, the tool's surface should be as smooth as possible, imposing constraints on the type of printer and nozzles suitable for production.

As the Si_3_N_4_ windows are highly fragile, the funnel's mouth must be large enough to encircle the window without touching the window itself. The tool must remain stable when placed on the MEMS chip, maintaining its position throughout use. To ensure this, the design has two broad legs next to the funnel. The legs and the funnel's mouth are aligned on the same plane to ensure the tool sits perfectly flat on the MEMS chip.

**Fig. 4:**
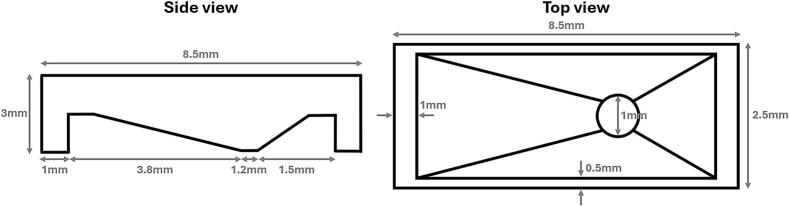
3D model for the MDT.

The edges, legs, and overall structure of the MDT are sufficiently thick to allow handling with tweezers without bending under the applied force. The testing was done on DensSolutions Climate chips, which have an asymmetric design, therefore also the MDT was designed asymmetrically, which can easily be adapted using the blend- or.stl-design in the Supplementary Information. For example, the leg closest to the inlet/outlet is positioned far enough away from the window to prevent any liquid that could spill underneath the funnel’s mouth from adhering to it, as this could potentially contaminate the inlet/outlet. Fig. 4 shows a simple technical drawing of the device with the dimensions we adapted to the MEMS chip used for testing.

The MDT therefore:

• Aims to enable localized dropcasting on MEMS based *in situ* chips for TEM studies.
• Is cost-effective and can be efficiently manufactured using a 3D printer.
• Is versatile and can be customized to suit specific experimental requirements.
• Is user-friendly and designed for ease of use.
• Minimizes failed *in situ* TEM experiments by reducing leaks and broken Si_3_N_4_ windows, thereby lowering overall experimental costs.

## 3 Design files summary

**Table:** 

**Design file name**	**File type**	**Open source license**	**Location of the file**
MDT_design	.stl-file	CC BY-NC 4.0	Available with the article
MDT_design	.blend-file	CC BY-NC 4.0	Available with the article

The MDT_design.stl- and MDT-design.blend-files include the design file that can be used as a the standard MDT design. This design can be adjusted according to the experimental needs. After this, this file can be used to 3D print the tool.

## 4 Bill of materials summary

**Table:** 

**Designator**	**Component**	**Number**	**Cost per unit −currency**	**Total cost −** **currency**	**Source of materials**	**Material type**
MDT_design	MDT	1	Cost depending on the 3D printer and chosen material, but roughly € 0.25.	Cost depending on the 3D printer and chosen material, but roughly € 0.25.	Depending on material used.	Other

## 5 Build instructions

• Adapt the design of the MDT (MDT_design.stl or MDT-design.blend) to the design of the available *in situ* MEMS chip
• Select the appropriate material for 3D printing based on the available printer and experimental parameters, including the solvent used. Ensure that the chosen material is chemically compatible and does not react with the solvent. During our testing, we used PLA, PETG, and Phrozen Aqua 8 K 3D printing resin in combination with common solvents such as water, ethanol, acetone, and dimethylformamide. All material–solvent combinations yielded positive results. A more comprehensive list of chemicals and compatible materials is available in [22].
• Print the MDT using the available 3D printer.
• The parameters listed below were used for our prints, but they are specific to our 3D printers and may vary depending on the printer available
  o The FDM prints where done using a Pruse MK2.5. Layer height was chosen at 0.07 mm with a nozzle of 0.25 mm. The printing was performed with the large top opening on the bed and the legs and funnel mount pointed up.
  o The SLA prints where done on two different printers: a Formlabs Form 3 and a Phrozen 8 k printer. Layer heights where chosen to be 0.05 mm. The printing was performed with the large top opening on the bed with supports only on that top edge.

## 6 Operation instructions

• Put the MEMS chip on a horizontal surface.
• Use tweezers to place the MDT on top of the MEMS chip, ensuring that the funnel mouth properly aligns around the Si_3_N_4_ window, as shown in Fig. 4. Avoid positioning it directly on the window, as any surface irregularities at the bottom of the MDT may cause damage.
• Use a pipette to dropcast a droplet of the sample solution into the MDT. The droplet size (µl) will vary based on experimental parameters such as solution density, particle size, and other relevant factors.
• Wait for the droplet to dry.
• Use tweezers to carefully remove the MDT from the MEMS chip, ensuring only vertical movement. Avoid any horizontal displacement, as it may damage the Si_3_N_4_ window.

## 7 Validation and characterization

Optical images where taken on a Tagarno FHD ZIP camera, while higher resolution optical microscopy images were taken on a Leica DMI5000M light microscope. Scanning electron microscopy (SEM) images were taken on a ThermoFisher (Eindhoven, Netherlands) Quanta 250 FEG, operated at 30 kV. To avoid charging during the SEM investigation, a 10 nm amorphous carbon coating was applied to the MDT using a Leica EM ACE600 coater. 3D models for the MDT were created using Blender.

For printing the MDT, several 3D printers and materials were tested: Ultimaker 3 extended 3D printer using polytactic acid (PLA), a stereolithography (SLA) 8 K resin printer, a fused deposition modeling (FDM) printer with polyethylene terephthalate glycol (PETG), using a 0.25 mm nozzles and 0.7 mm layer thickness and an SLA printer utilizing a Formlabs laser system. Dropcasting was performed using a 10 µl Eppendorf 4,924,000,029 reference micropipette, set to disperse 3 µl droplets.

**Fig. 5:**
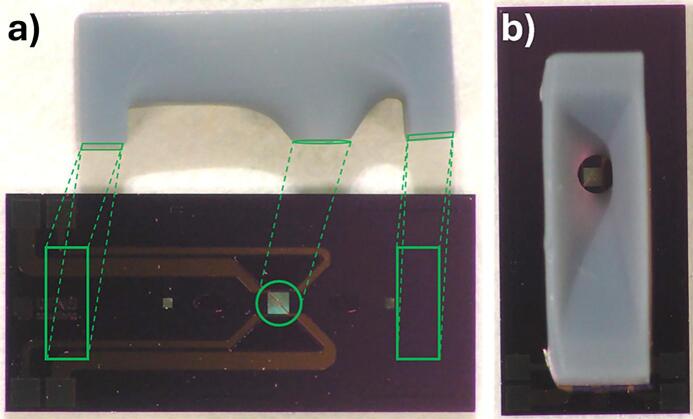
a) Size comparison of the finalized MDT next to a MEMS *in situ* chip. The intended placement of the tool is indicated on the figure. b) MDT placed on top of the MEMS chip. The mouth of the tool fits just around the Si_3_N_4_ window.

Fig. 5 illustrates the finalized tool alongside the MEMS chip used for testing. This tool was fabricated using an SLA 8 K resin 3D printer and had a 0.5 mm funnel opening. Based on 10 different tries for dropcasting for each tool, this combination produced the most consistent results. As shown in Fig. 4b, the tool’s mouth encircles the Si_3_N_4_ window, preventing damage, and the chip’s asymmetry has been fully integrated into the design, placing the funnel’s mouth more towards one leg of the tool. With this setup, the tool costs 0.25 € per piece, making it easily replaceable.

**Fig. 6:**
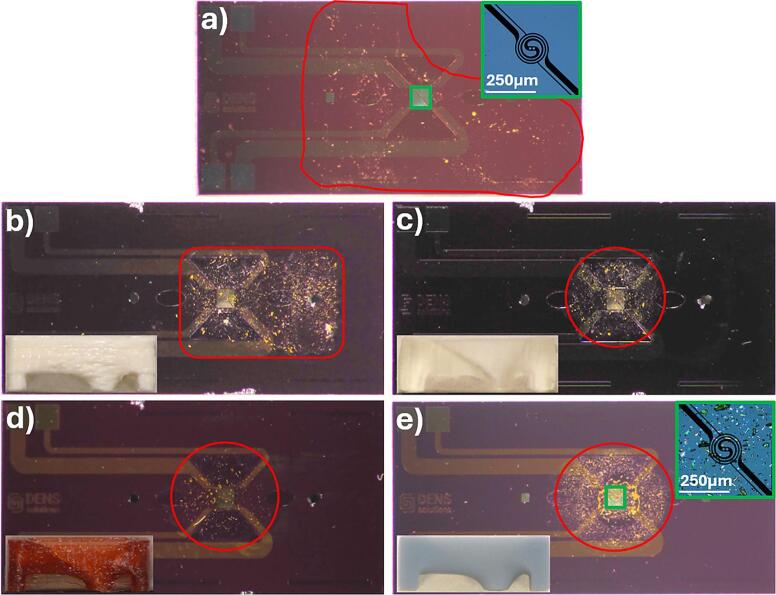
Images of an *in situ* chip with the printed tool as an inset, showcasing the particle distribution after dropcasting a droplet of the testing solution: a) without the MDT, b) using an Ultimaker 3 Extended 3D printer with PLA material, c) using an SLA printer with a Formlabs laser system, d) using an FDM printer with PETG, utilizing a 0.25 mm nozzle and 0.7 mm layer thickness, and e) using an SLA 8 K resin 3D printer. For panels a) and e), additional insets outlined in green provide magnified views of the Si_3_N_4_ window. The edges of the droplets are marked in red, and particles found outside these boundaries were identified as contamination resulting from attempts to clean the chip post-dropcasting. (For interpretation of the references to colour in this figure legend, the reader is referred to the web version of this article.)

Several 3D printer types and materials were tested to identify the optimal combination for dropcasting controlled-size droplets onto MEMS chips. Fig. 6 compares the results of dropcasting a 3 µl droplet of a solution containing ethanol and MOF-74 particles, with the MDT printed using several different 3D printers and compares it to the result of dropcasting without the MDT. For most of the 3D printers tested, except for Ultimaker 3, the droplet confined by the tool remains within the mouth area, avoiding contamination of the O-ring and the inlet/outlet holes. Fig. 6 also provides a higher magnification view of the Si_3_N_4_ window area with and without the tool (SLA 8 K resin 3D printer). It clearly demonstrates that the use of the tool results in a greater concentration of particles on the windows, improving particle placement efficiency. To assess the tool’s effectiveness, a dense ethanol/MOF-74 solution with micrometer-sized MOF-74 particles was dropcasted 10 times with (SLA 8 K resin printer) and without the tool. The high particle density increased the likelihood of particles settling on the windows without the tool. On average, 3 ± 2) particles were observed on the window without the tool, compared to 12 ± 5 with the tool. However, due to the solution’s density, particle agglomerates often fully covered one or more windows during tool-assisted dropcasting, making exact counting impossible. Only individually distinguishable particles were counted, meaning the actual number was higher.

We observed that some liquid spreads underneath the tool; however, due to surface tension and the small volume of liquid, it adheres to the underside and outer edge of the MDT mouth, preventing further spreading. This behavior is likely caused by minute irregularities on the underside of the tool’s mouth. Because of this spreading, it is important that the wall of the tool’s mouth should be as thin as possible and thus the area of contact between the tool and the chip as small as possible. With a larger contact area, the fluid spreads to the larger area underneath the tool’s mouth.

**Fig. 7:**
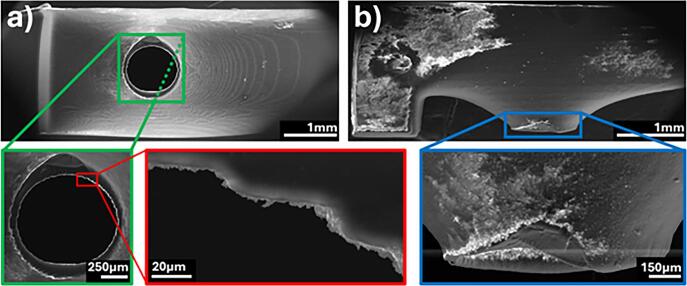
SEM images of the MDT printed using the SLA 8 K resin 3D printer in a) top-down and b) side views.

To investigate further, SEM images of this region were taken from the tool printed using the SLA 8 K resin 3D printer (Fig. 7). These images confirm the presence of irregularities on the underside of the tool’s mouth. Such imperfections likely disrupt the surface tension of the liquid, allowing it to pass through gaps and counteracting the capillary forces that would otherwise keep the droplet contained. However, as demonstrated, even with these irregularities that were due to the use of a common 3D printer, the droplet still was confined to the targeted area.

Lower-end 3D printers may have reduced accuracy, which could introduce sharp edges or irregularities that may lead to window breakage or suboptimal results. Although we have not quantitatively measured the relationship between surface roughness and tool performance, our qualitative observations indicate that smoother surfaces improve liquid guidance into the funnel and enhance droplet containment. For example, the SLA 8 K resin 3D printer produced the most consistent results, while the Ultimaker 3 Extended 3D printer with PLA showed the poorest performance (see Fig. 6). This effect is likely due to surface irregularities creating small gaps under the funnel walls, which allow liquid to escape. In contrast, flatter surfaces help keep the droplet confined. While all 3D-printed parts exhibit some surface roughness, minimizing these irregularities appears to improve overall tool performance.

**Fig. 8:**
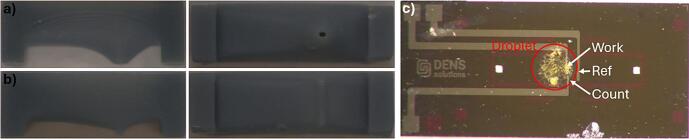
MDT’s with an a) smaller and b) rectangular shaped funnel mouth, intended to be used for liquid biasing chips. c) Picture of an *in situ* liquid biasing chip. The droplet edges, as well as the reference, working and counter electrodes have been indicated on the image.

Some *in situ* MEMS chips, particularly those used in liquid experiments involving biasing, feature smaller, rectangular windows. These experiments often require particles to be deposited only on the working electrode, avoiding the counter and reference electrodes. To address this, we adjusted the tool’s design by either reducing the diameter of the funnel mouth or modifying its shape to a rectangular one. (Fig. 8) While this approach successfully prevented particles from depositing on the reference electrode, we could not avoid deposition on the counter electrode. For the circular, funnel-shaped mouth, the reduced size (0.2 mm) of the funnel mouth often caused droplets to become trapped due to surface tension, preventing them from passing through. In the case of the rectangular-shaped funnel, no liquid was able to pass through. Therefore, there is a lower limit to the size of the funnel opening. While the 0.2 mm opening did not allow liquid to pass through, the 0.5 mm opening of the funnel mouth, used for all other MDT’s discussed in this paper, consistently allowed for successful liquid passage. Although we have not yet succeeded in adjusting the MDT for liquid-biasing chips, further design modifications—particularly to address surface tension issues—may yield better results in the future. One possible solution to address the surface tension issue is plasma cleaning the device prior to dropcasting in order to render the surface hydrophilic. Although initial tests have shown promising results, further investigation is required.

The MDT simplifies sample preparation for MEMS-based *in situ* TEM holders. The MDT can be readily produced using 3D printing and is easy to use. By confining the dropcasted droplet to a specific area, the tool prevents particles in the solution from migrating under the O-ring between the bottom and top chips, thus reducing the chances for leaks, and prevents contamination of the holder’s inlet and outlet ports by the particles. By containing the droplet, the tool increases the likelihood that sample particles are positioned on the electron-transparent window, which reduces the volume of solution needed for dropcasting. These advantages make the MDT a practical asset for both time management and cost reduction in MEMS-based TEM experiments. This MDT was designed specifically for MEMS chips from one manufacturer, but its dimensions can be easily adjusted to accommodate MEMS chips from other manufacturers with varying designs.

## Ethics statements

The authors declare no conflict of interest. The funders had no role in the design of the study; in the collection, analyses, or interpretation of the data; in the writing of the manuscript, or the decision to publish the results.

## CRediT authorship contribution statement

**Matthias Quintelier:** Writing – review & editing, Writing – original draft, Validation, Methodology, Investigation, Formal analysis, Data curation, Conceptualization. **Joke Hadermann:** Writing – review & editing, Writing – original draft, Validation, Supervision, Resources, Methodology, Formal analysis, Conceptualization.

## Declaration of competing interest

The authors declare that they have no known competing financial interests or personal relationships that could have appeared to influence the work reported in this paper.
