# Supplementary material for: MDT: A simple tool to facilitate dropcasting on in situ TEM MEMS chips
Source: HardwareX. 2025 Jun 21;23:e00666. doi: 10.1016/j.ohx.2025.e00666 (PMC12226395; doi:10.1016/j.ohx.2025.e00666)
Supplement: Supplementary Data 1 [file mmc1.docx]

Design Files

The design of the MDT:


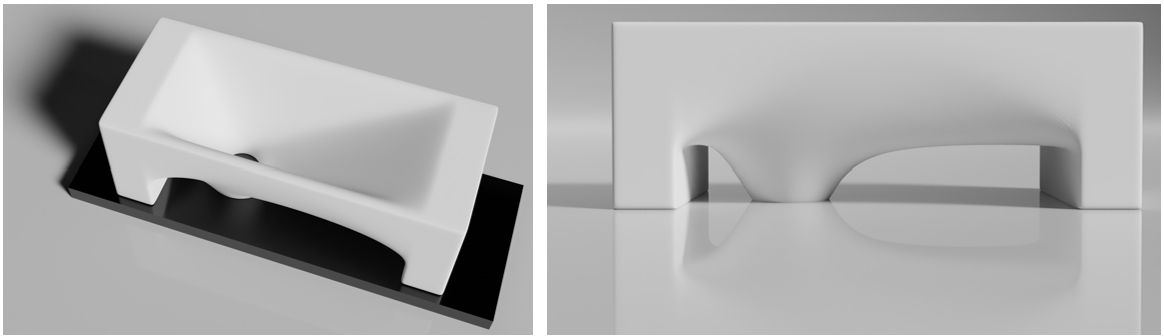


The .stl file used for printing (MDT_design.stl) is included in the submission.
